# Use of thoracic electrical impedance tomography as an auxiliary tool for alveolar recruitment maneuvers in acute respiratory distress syndrome: case report and brief literature review

**DOI:** 10.5935/0103-507X.20150068

**Published:** 2015

**Authors:** Regis Goulart Rosa, William Rutzen, Laura Madeira, Aline Maria Ascoli, Felippe Leopoldo Dexheimer Neto, Juçara Gasparetto Maccari, Roselaine Pinheiro de Oliveira, Cassiano Teixeira

**Affiliations:** 1 Department of Intensive Care Medicine, Hospital Moinhos de Vento - Porto Alegre (RS), Brazil.

**Keywords:** Cardiography, impedance, Respiratory insufficiency/physiopathology, Monitoring, physiologic, Respiratory mechanics, Case reports

## Abstract

Thoracic electrical impedance tomography is a real-time, noninvasive monitoring tool of the regional pulmonary ventilation distribution. Its bedside use in patients with acute respiratory distress syndrome has the potential to aid in alveolar recruitment maneuvers, which are often necessary in cases of refractory hypoxemia. In this case report, we describe the monitoring results and interpretation of thoracic electrical impedance tomography used during alveolar recruitment maneuvers in a patient with acute respiratory distress syndrome, with transient application of high alveolar pressures and optimal positive end-expiratory pressure titration. Furthermore, we provide a brief literature review regarding the use of alveolar recruitment maneuvers and monitoring using thoracic electrical impedance tomography in patients with acute respiratory distress syndrome.

## Abstract (pt)

A tomografia por impedância elétrica torácica constitui ferramenta de monitorização não invasiva, em tempo real, da distribuição regional da ventilação pulmonar. Sua utilização à beira do leito em pacientes com síndrome do desconforto respiratório agudo tem o potencial de auxiliar na condução de manobras de recrutamento alveolar, frequentemente necessárias em casos de hipoxemia refratária. Neste relato de caso, apresentamos os resultados e a interpretação da monitorização da tomografia por impedância elétrica torácica em um paciente com síndrome do desconforto respiratório agudo, durante manobras de recrutamento alveolar, com aplicação transitória de altas pressões alveolares e titulação da pressão positiva ao final da expiração ideal. Adicionalmente, apresentamos uma breve revisão da literatura a respeito do uso de manobras de recrutamento alveolar e monitorização com tomografia por impedância elétrica torácica em pacientes com síndrome do desconforto respiratório agudo.

## INTRODUCTION

Acute respiratory distress syndrome (ARDS) is characterized by acute onset respiratory failure associated with severe hypoxemia (oxygen partial pressure/fraction of inspired oxygen - PaO_2_/FiO_2_ ≤ 300mmHg) and bilateral pulmonary infiltrates not fully explained by heart failure or fluid overload.^(1)^ According to the Berlin criteria, ARDS may be classified as mild (PaO_2_/FiO_2_ ranging from 201 to 300mmHg), moderate (PaO_2_/FiO_2_ ranging from 101 to 200mmHg) or severe (PaO_2_/FiO_2_ ≤ 100mmHg).^(2)^ ARDS continues to be correlated with high mortality rates, which may reach 36% to 44% in specialized centers, despite the progress made in treatment in recent decades.^(3,4)^

Patients with ARDS often require intensive treatment using mechanical ventilation (MV), given the severity of their respiratory failure. However, MV may have deleterious effects on lung tissue and may even contribute to worsening of ARDS.^(5-7)^ This deleterious effect was demonstrated in studies evaluating the impact of MV with high tidal volumes and plateau pressure levels.^(8,9)^ Therefore, MV adjustment is recommended to avoid or minimize ventilator-associated lung injuries.^(10-12)^ This goal must be dynamically reached and should be based on information from the monitoring of the pulmonary function and respiratory mechanics of patients with ARDS.

The use of alveolar recruitment maneuvers (ARM) has been proposed as complementary therapy to ventilation strategies for patients with severe ARDS and refractory hypoxemia.^(13,14)^ Those maneuvers aim to expand collapsed alveoli using a transient increase in transpulmonary pressure and by subsequently applying adequate positive end-expiratory pressure (PEEP) to prevent alveolar derecruitment. This strategy aims to strike an adequate balance between the number of collapsed and hyperextended alveoli, which reduces the pulmonary shunt and therefore improves the ventilation/perfusion ratio and hypoxemia.^(15)^

The bedside use of thoracic electrical impedance tomography (EIT) in patients with ARDS may be a key auxiliary tool for ARM by enabling clinicians to choose an optimal PEEP for maximum recruitment after considering key variables, including pulmonary static compliance (Cst), recruitable alveolar collapse and alveolar overdistension. That examination is based on existing differences in electrical properties generated by changes in air content in small lung areas, which create an impedance relationship between such areas.^(16)^ The pixels generated in the image of the monitor represent changes in the percentage of local impedance compared with a reference assessed in the beginning of the image acquisition. Thus, the dynamic image of the thoracic EIT monitor shows local air change during ventilation in real time. Color variation in the generated image shows areas within the alveoli that undergo air changes, with a scale ranging from dark blue (lower aeration) to light blue (higher aeration). Grey images represent areas in which no aeration change occurred.^(16)^ During the decremental PEEP titration maneuver, alveolar collapse may occur in specific lung areas (causing reduced Cst), and alveolar overdistension relief may occur in other lung areas (causing increased Cst). The degree of recruitable alveolar collapse is estimated by the reduction in pixel compliance, in relation to its best compliance, caused by the reduced PEEP value. Conversely, alveolar overdistension represents the reduction in pixel compliance, in relation to its best compliance, caused by increased PEEP.

In the present case report, we describe the application of thoracic EIT in a patient with severe ARDS during ARM with transient application of high alveolar pressures and optimal PEEP titration.

## CASE REPORT

**Figure 1:**
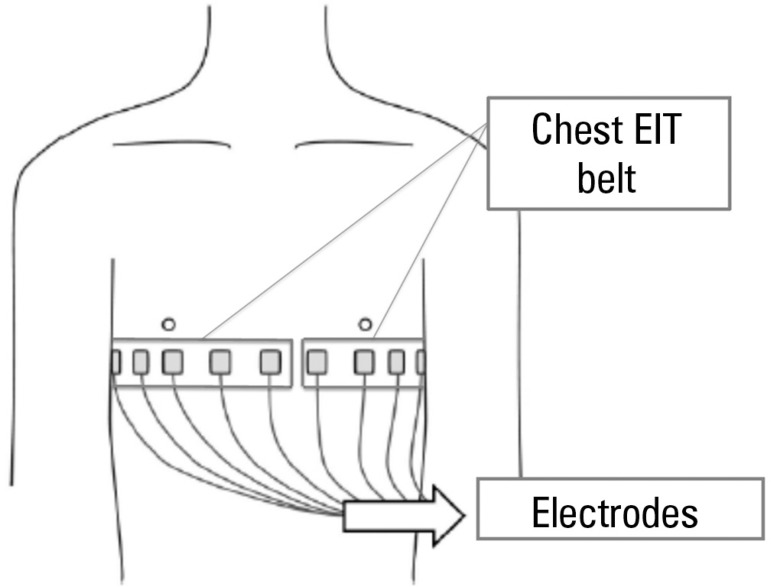
Positioning of the electrodes for thoracic electrical impedance tomography. The electrodes for thoracic electrical impedance tomography are attached to the chest wall using a strap placed across the chest, near the level of the mammary line (between the fourth and fifth intercostal spaces). A flow sensor positioned between the endotracheal tube and the "Y" of the ventilator circuit is also connected to the monitor for electrical impedance tomography, in addition to the electrocardiogram electrodes (not shown in this Figure). EIT - electrical impedance tomography.

**Figure 2:**
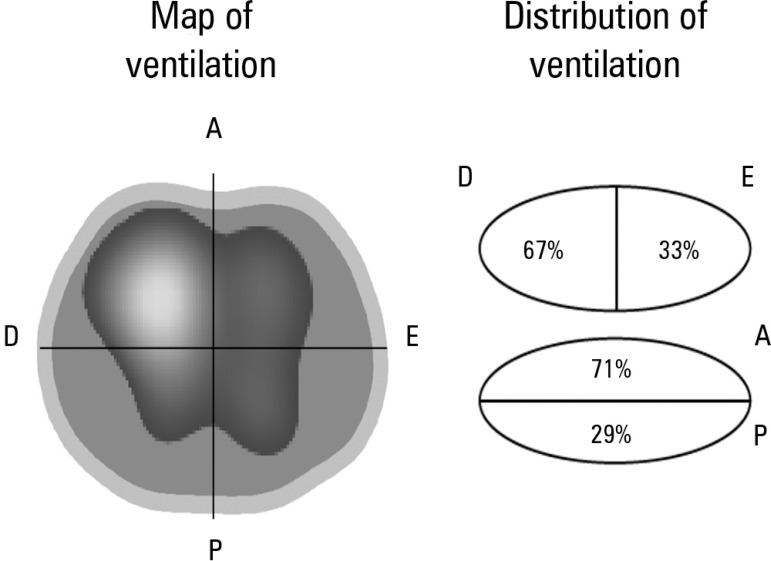
Thoracic electrical impedance tomography: distribution of pulmonary ventilation before the alveolar recruitment maneuver. Image of a chest cross-section (at the level of the electrode position). An asymmetric distribution of alveolar ventilation is noted with reduced distribution of ventilation in the left lung and in posterior lung fields. R - right; L - left; A - anterior; P - posterior.

**Figure 3:**
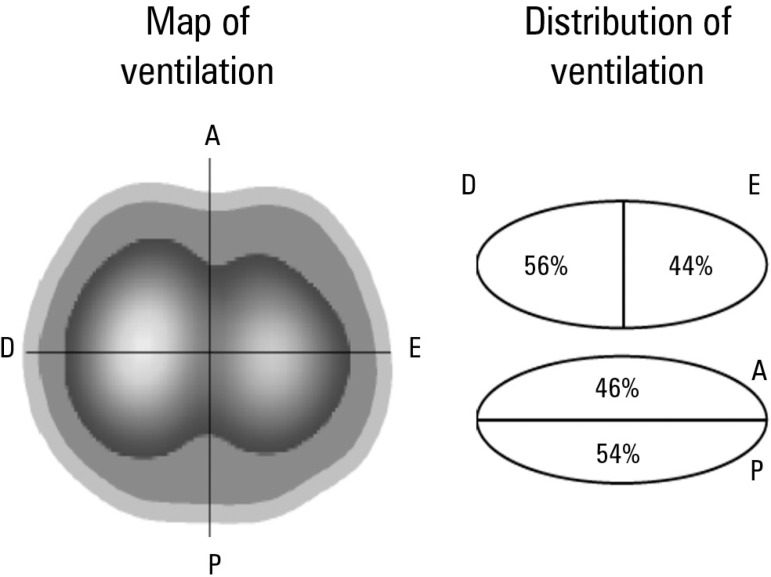
Thoracic electrical impedance tomography: distribution of pulmonary ventilation after the alveolar recruitment maneuver. Image of a chest cross-section (at the level of the electrode position). A more homogeneous distribution of alveolar ventilation is noted in comparison with Figure 2. A higher ventilation of previously underventilated lung areas (left lung and posterior lung fields) occurred. R - right; L - left; A - anterior; P - posterior.

**Figure 4:**
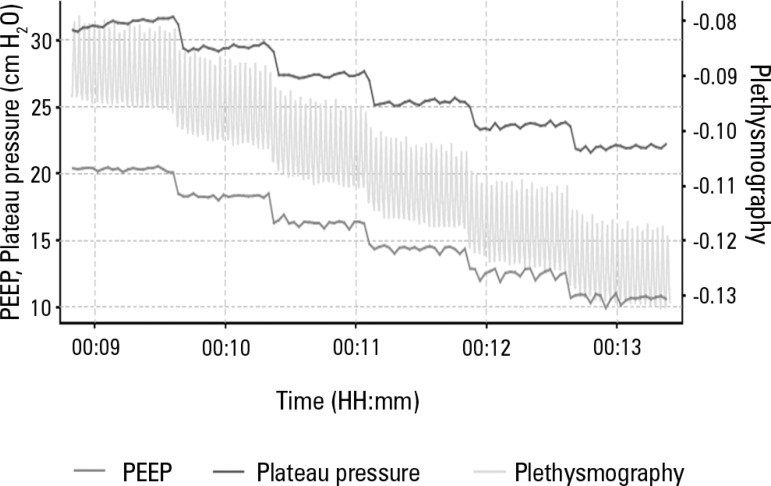
Graphical representation of decremental positive end-expiratory pressure titration after the alveolar recruitment maneuver. Graphical representation of the plateau pressure performance and pulmonary plethysmography during the decremental positive end-expiratory pressure titration. PEEP - positive end-expiratory pressure.

**Figure 5:**
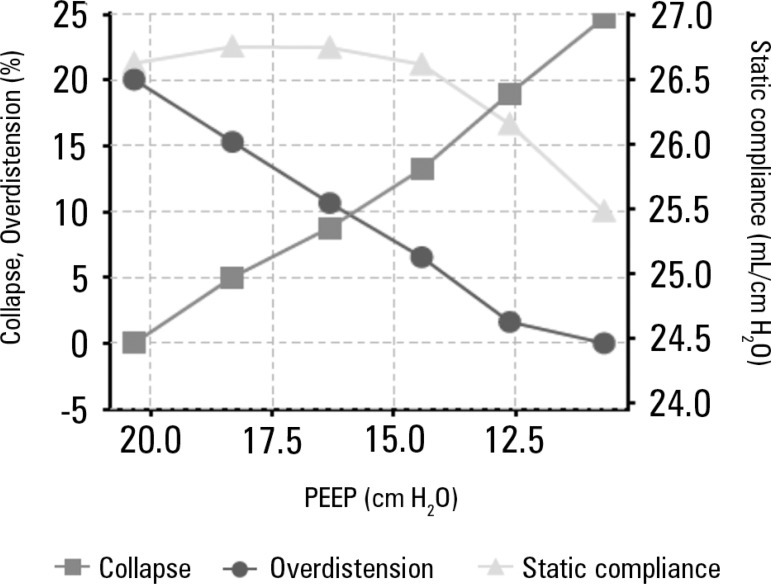
Ratios between static compliance, alveolar collapse and alveolar overdistension according to the positive end-expiratory pressure applied. Graphical representation of the values of lung static compliance, alveolar collapse index and alveolar overdistension index for each positive end-expiratory pressure value. Note the decrease in alveolar overdistension index and increase in alveolar collapse index with the progressive decrease in positive end-expiratory pressure. The values of lung static compliance have a nonlinear correlation with the positive end-expiratory pressure, producing a more pronounced decrease in lung static compliance for positive end-expiratory pressure values lower than 15cmH_2_O in this case. PEEP - positive end-expiratory pressure.

**Figure 6:**
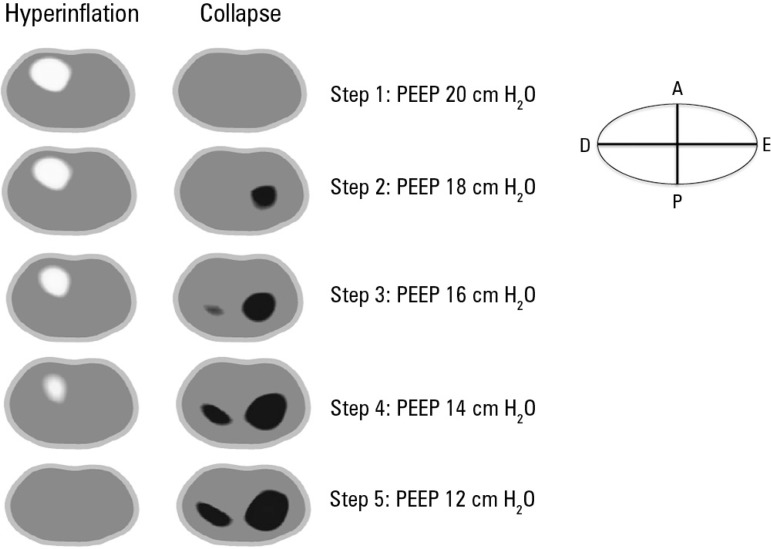
Thoracic electrical impedance tomography: alveolar collapse and overdistension areas according to the positive end-expiratory pressure applied. Graphical representation of the sites of occurrence of alveolar overdistension and collapse for each positive end-expiratory pressure applied. The hyperdistended areas are shown in white. The collapsed areas are shown in dark blue. The occurrence of alveolar overdistension is noted in anterior right lung fields for higher values of positive end-expiratory pressure. The occurrence of alveolar collapse is noted, especially in the left lung and posterior lung fields, for lower values of positive end-expiratory pressure. PEEP - positive end-expiratory pressure; R - right; L - left; A - anterior; P - posterior.

A 63-year-old Caucasian male patient with a diagnosis of liver cirrhosis caused by nonalcoholic steatohepatitis was admitted to the intensive care unit (ICU) of a tertiary hospital for severe community-acquired pneumonia. Upon hospital admission, the patient had fever, productive cough and dyspnea. Chest X-ray showed an initial focus of bronchopneumonic consolidation in the right lower lobe. The patient developed hypoxic respiratory failure associated with worsened pulmonary radiological condition, showing extensive bilateral alveolar consolidations, even after initiation of antibiotic therapy using piperacillin/tazobactam and clarithromycin, hydration and respiratory support with oxygen therapy by nasal cannula. Bedside transthoracic echocardiogram showed normal systolic function of the left and right ventricles. Following the diagnosis of ARDS, the patient was managed with continuous parenteral sedoanalgesia, neuromuscular block and protective MV with a tidal volume of 6 mL/kg predicted weight and PEEP/FIO_2_ ratio according to the Acute Respiratory Distress Syndrome Network (ARDSnet) protocol.^(9)^ However, severe hypoxemia (112mmHg PaO_2_/FiO_2_) and high alveolar pressures (34cmH_2_O plateau pressure) persisted, even after MV optimization. At that time, the patient was monitored using a Timpel Enlight 1800^®^ (São Paulo, Brasil) thoracic electrical impedance tomograph (Figure 1), which showed asymmetric ventilation distribution (Figure 2), highlighting the hypoventilation of the left lung and posterior lung fields. Alveolar recruitment with transient application of high alveolar pressures, using the pressure-controlled ventilation mode (40cmH_2_O PEEP, 20cmH_2_O pressure above PEEP and 1:1 inspiratory/expiratory time ratio) for 2 minutes was used after normovolemia was confirmed using ultrasonographic evaluation of the inferior cava vein variability. No hemodynamic instability occurred during the process. The analysis of the thoracic EIT following ARM (Figure 3) showed improved lung ventilation distribution compared with the initial examination, highlighting the increased ventilation in previously collapsed areas. Following ARM, the optimal PEEP was calculated using the ratios between PEEP, Cst and alveolar collapse and overdistension indices (Figures 4, 5 and 6). Decremental PEEP titration was performed in 2-cmH_2_O steps. The PEEP associated with the highest Cst values and lowest alveolar collapse and overdistension values was chosen. Based on the analysis of the plot generated by the thoracic EIT, 16cmH_2_O PEEP was deemed optimal in this situation because it showed the best ratio between Cst and alveolar collapse and overdistension; values lower than 16cmH_2_O were associated with lower Cst values and higher lung collapse values, and PEEP values higher than 16cmH_2_O were associated with higher values of alveolar overdistension. Then, the patient was recruited again, as described above, and the ventilatory parameters were maintained with a tidal volume of 6mL/kg predicted weight and 16cmH_2_O PEEP. At this time, the plateau pressure measured was 26cmH_2_O, and the PaO_2_/FiO_2_ ratio was 226mmHg.

## DISCUSSION

Alveolar recruitment may be defined as a strategy that transiently increases alveolar pressure above the regular tidal ventilation, including (but not limited to) sustained inflation maneuvers, gradual PEEP increase, tidal volume increase or controlled pressure and extended sighs. Alveolar recruitment aims to improve gas exchange through maximum recruitment of alveolar units, providing more homogeneous ventilation of the lung parenchyma. The ARM described in the case reported, with subsequent PEEP titration to maintain open lungs with an optimal ratio between alveolar collapse and overdistension, was initially evaluated by computerized tomographic analysis.^(17)^ This strategy aims to open the lungs early and to keep them open, as initially advocated by Lachmann,^(18)^ in order to achieve improved lung function and avoid a potential lung injury associated with MV.^(19)^

Because ARDS is a biphasic disease that progresses from an acute exudative phase to an organization phase with persistent alveolar and interstitial fibrosis, early diagnosis, preferably within 72 hours, is crucial for effective ARM and for maintenance of alveolar opening with sufficient PEEP.^(20-23)^ A study analyzing 85 patients with ARDS classified according to the extent of fibroproliferation in the computed tomography scan showed that higher computed tomography scores were associated with a significant decrease in days free of organ dysfunction and days free of MV; higher scores were an independent risk factor for mortality (odds ratio: 1.2; 95% confidence interval [CI]: 1.06 - 1.36; p < 0.005).^(24)^ A recent meta-analysis^(25)^ of 10 randomized clinical trials that evaluated the effects of ARM on patients with ARDS showed a risk ratio (RR) of 0.84 (95% CI: 0.74 - 0.95) for in-hospital mortality. However, the quality of evidence was low, given the risk of bias in the included studies, as ARM was usually performed in conjunction with other ventilatory interventions that may have affected the outcome of interest. In the present study, no differences were observed in the barotrauma rates (RR: 1.11; 95% CI: 0.78 - 1.57) or in the need for rescue therapies (RR: 0.76; 95% CI: 0.41 - 1.40). Most studies failed to show any difference between groups regarding the duration of MV and the length of ICU or hospital stay. Ongoing studies may best assess whether ARM should be routinely applied to optimize the clinical outcomes of patients with ARDS.^(26,27)^

EIT is a noninvasive method of bedside monitoring that provides real-time information on the regional distribution of changes in the electrical resistivity of the lung tissue resulting from ventilation or blood flow (perfusion) variations in relation to a predetermined reference.^(28,29)^ Data provided by EIT may directly quantify local pulmonary impedance changes. Therefore, functionally active pulmonary structures are shown, while functionally static, normal or pathologically structures (for example, pleural or pneumothorax effusion) are not captured by the method and are not imaged. Several animal and human studies have validated the pulmonary findings of thoracic EIT.^(30,31)^ However, that linear correlation fundamentally depends on the position of the electrode, changes in the thoracic wall and diaphragm shape and the ratio of tidal ventilation distributed in lung areas.^(32)^

The definition of collapsed and overdistended lung areas may enable thoracic EIT to be used as a bedside clinical tool for adjusting the MV parameters. When calculating the potentially recruitable lung volume as the difference between the open, totally recruited and non-recruited lung volume at 40cmH_2_O, Lowhagen et al.^(33)^ observed a significant value of potentially recruitable lung volume of 26 ± 11% (11% to 47%) in patients with ARDS. The same group also analyzed the regional distribution of intratidal gas upon changes in regional lung mechanics using EIT data and airway pressure at different PEEP levels.^(34)^ The tidal volume was primarily distributed in the medio-ventral areas, although the increase in PEEP caused redistribution into the more dorsal areas, regardless of the PEEP level. A recent clinical study showed that thoracic EIT performed immediately above the diaphragm could detect alveolar derecruitment during a decremental PEEP maneuver, both in dependent and non-dependent lung areas, and also show alveolar collapse.^(35)^ Recently, Costa et al.^(36)^ evaluated a new algorithm to estimate hyperinsuflation during a decremental PEEP maneuver and showed that the hyperinsuflation lung areas were particularly similar when comparing EIT with CT, although EIT systematically had a higher overdistension coefficient. The authors concluded that the EIT estimates of hyperinsuflation mostly account for the functional deterioration of pulmonary units, rather than anatomic changes of those areas.

## CONCLUSION

The bedside use of thoracic electrical impedance tomography may be a clinical tool that is able to guide, at each breath, possible adjustments of regional ventilation, including the decision for alveolar recruitment maneuvers, in patients with acute respiratory distress syndrome. Furthermore, electrical impedance tomography may refine the choice of optimal post-recruitment positive end-expiratory pressure, considering the quantification of possibly deleterious variables, including alveolar collapse and overdistension. Future studies are necessary to evaluate the use of this device combined with software to help identify situations in which mechanical ventilation could be optimized.
